# Tea and Coffee

**Published:** 1881-10

**Authors:** 


### TEA AND COFFEE.

There are certain districts in the United States where new notions of every description flourish with amazing vigor, as far as the number of converts are concerned; among these mere notions are the injurious effects of tea and coffee as a daily drink.

I think that it is demonstrable that a single cup of weak tea or coffee at a meal, especially in cold weather, and most especially in persons of a weakly habit or constitution, is far more healthful than a glass of cold water.

Tea and coffee doubtless do injure some people—that is, some persons may not be able to drink them without its being followed by some discomfort; so will even water, if used too freely; and I think it will be found that, in nearly every such case of uncomfortableness after a cup of tea or coffee, this condition of things has been brought about by the too free use of these articles, or that the tone of the stomach has been impaired by improper eating.

Man is styled an omnivorous animal—an animal eating everything. No created animal can eat and drink, without discomfort, half the articles consumed by man. I know very well that men die before their days are half numbered, in consequence of errors in eating and drinking; but these disastrous results do not arise from the quality of man's aliment, but from its quantity—it is the quantity which prematurely kills millions. A sensible man may eat almost anything with impunity, a simpleton nothing; the former eats like a philosopher, the latter like a pig. The former eats as much as he wants, the latter eats more than he wants.

In small quantities, and occasionally, many things may be eaten with advantage, which, if eaten continuously for weeks and months, or in inordinate amounts, would occasion serious results. There are also times and seasons for different articles of food; for example: fruits and berries, when ripe, fresh, perfect, may be freely eaten in the earlier parts of the day, but if largely eaten after sundown, especially at some seasons of the year, actually endanger life, and have destroyed thousands.

